# Novel Concrete Temperature Monitoring Method Based on an Embedded Passive RFID Sensor Tag

**DOI:** 10.3390/s17071463

**Published:** 2017-06-22

**Authors:** Yongsheng Liu, Fangming Deng, Yigang He, Bing Li, Zhen Liang, Shuangxi Zhou

**Affiliations:** 1 School of Civil Engineering and Architecture, East China Jiaotong University, Nanchang 330013, China; 2530@ecjtu.jx.cn (Y.L.); zhoushuangxi@ecjtu.jx.cn (S.Z.); 2 School of Electrical and Automation Engineering, East China Jiaotong University, Nanchang 330013, China; 3 School of Electrical Engineering and Automation, Hefei University of Technology, Hefei 230009, China; heyigang1966@mail.hfut.edu.cn (Y.H.); libinghnu@hfut.edu.cn (B.L.); 4 Rising Micro Electronics Co., Ltd., Guangzhou 510006, China; zliang@rising-ic.com

**Keywords:** temperature sensor, radio frequency identification (RFID), concrete temperature measurement

## Abstract

This paper firstly introduces the importance of temperature control in concrete measurement, then a passive radio frequency identification (RFID) sensor tag embedded for concrete temperature monitoring is presented. In order to reduce the influences of concrete electromagnetic parameters during the drying process, a T-type antenna is proposed to measure the concrete temperature at the required depth. The proposed RFID sensor tag is based on the EPC generation-2 ultra-high frequency (UHF) communication protocol and operates in passive mode. The temperature sensor can convert the sensor signals to corresponding digital signals without an external reference clock due to the adoption of phase-locked loop (PLL)-based architecture. Laboratory experimentation and on-site testing demonstrate that our sensor tag embedded in concrete can provide reliable communication performance in passive mode. The maximum communicating distance between reader and tag is 7 m at the operating frequency of 915 MHz and the tested results show high consistency with the results tested by a thermocouple.

## 1. Introduction

Temperature control for concrete plays a dominant role during its curing and maturation period [1]. The hydration heat generated during the pouring process of concrete may lead to large temperature differences between the inside and the outside of the concrete, which can easily cause cracks in concrete [2,3]. Thus, the concrete temperature should be well supervised during its pouring period to obtain an appropriate hydration heat releasing rate. Furthermore, the curing temperature determines the volume stability and the structural strength of concrete [4,5,6]. Uncontrolled temperature increases will result in increasing shrinkage stress, strength reduction, and structure ageing [7]. However, if the ashlar of fresh concrete could be heated in a controlled way, the curing time will be reduced and the productivity will be largely enhanced.

The traditional measuring techniques, such as thermocouple temperature measurement (TTM) [8,9,10] and fiber temperature detection (FTD) [11,12], are wired methods. The high cost and complex layout of TTM limit its wide application, so TTM is always employed for sample testing. FTD also requires high cost and complex layout; moreover, the fiber is easily broken, which requires regular maintenance. Along with the tremendous development of the Internet of Things, online monitoring techniques based on wireless sensor networks (WSN) have attracted significant attention, and this technique has been widely used in structural health monitoring (SHM) and fault early warning due to its high flexibility [13,14]. At present, the WSN node usually operates in active mode for a long operating range and high transmission rate. However, restricted by the battery power supply, the working life of a WSN node lasts only two or three years, hence, the cost of the WSN technique is still high for large-scale commercial applications. Recently, owing to the progress of radio frequency identification (RFID) and sensor fabrication technology, online monitoring systems based on RFID sensor tags has been a hot topic [15,16,17,18]. Compared with existing WSN techniques, the ultra-high frequency RFID sensor tag has a shorter operating range, but it has a simple structure, low power consumption, and can work in passive mode due to its back-scattering mechanism. Furthermore, the RFID sensor tag has a unique identification number, allowing an abnormal area to be quickly located. Therefore, the RFID sensor tag is especially suitable for long-term and low-cost monitoring applications, such as SHM application.

Various types of RFID temperature sensors have been already reported. The simplest type of RFID temperature sensors is the chipless type [19,20], which requires no integrated circuits (ICs) and transmits sensor data by changing the radar cross-section of the antenna. However, due to a lack of digital processing blocks, these chipless RFID sensors show low performance and are mainly applied in low-cost fields. The chip-based RFID temperature sensors can be divided into two groups: the printed circuit board (PCB)-based type [21,22] and the IC-based type [23,24,25]. The PCB-based RFID temperature sensors employs a microcontroller unit (MCU) to control various external sensors and the IC-based RFID temperature sensors integrate the temperature sensors with RFID tag chips. The temperature sensor can be easily fabricated using the standard CMOS process. Though PCB-based RFID sensors exhibit high flexibility and short designing time, IC-based RFID temperature sensors hold the advantages of lower power dissipation, smaller board area, and lower cost.

Our previous work proves the feasibilities of applying RFID sensors for concrete monitoring [26], however, that work just utilizes current commercial RFID sensor tags for measurements. Hence, systematic research should be conducted for further application. This work proposes a novel measuring method for concrete temperature monitoring based on an IC-based passive RFID sensor tag. The rest of the paper is organized as follows: Section 2 introduces the theory of intelligent temperature control; Section 3 analyzes electromagnetic wave transmission mechanisms in concrete; Section 4 shows the overall structure of the proposed sensor tag and, consequently, introduces the design of key blocks of the proposed RFID sensor; Section 5 shows the experimental results, which are also compared with the traditional measuring method; and Section 6 makes a conclusion.

## 2. Principles of Intelligent Temperature Control

**Figure 1:**
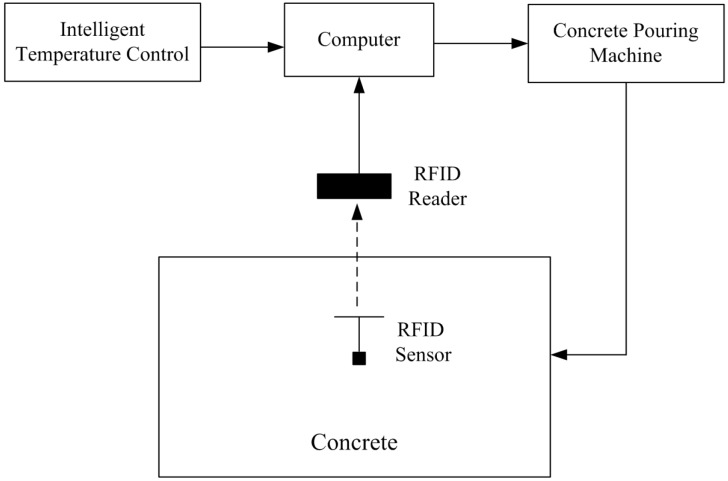
Proposed concrete-temperature monitoring method.

Figure 1 shows the architecture of our proposed concrete-temperature monitoring method based on an embedded passive RFID sensor tag. The RFID sensor tag is embedded in concrete for accurate temperature monitoring. The RFID reader received the temperature data from the embedded RFID sensor and then the computer controlled the concrete pouring machine based on the intelligent temperature control pattern discussed below.

In order to avoid concrete cracking during the concrete cooling process, there are three basic principles that should be considered, including maximum temperature control, the rate of temperature change control, and abnormal temperature control [27]. Firstly, large temperature differences between the internal and external of concrete can lead to concrete cracking, hence, the maximum internal temperature should be different according to the ambient temperature. Secondly, rapid temperature change can also cause the concrete to crack. Moreover, excessively high or low ambient temperature may also cause the concrete to crack. In these situations, a control system should be adopted to decrease the temperature stress.

Considering the internal heat source, a basic equation of three-dimensional heat conduction can be obtained according to the Fourier Law [28]:

(1) $$\frac{\partial T}{\partial \tau }=\left(\frac{\partial^{2}T}{\partial x^{2}}+\frac{\partial^{2}T}{\partial y^{2}}+\frac{\partial^{2}T}{\partial z^{2}}\right)+\frac{Q}{\rho c}$$

where *T* represents the concrete temperature, *τ* is the time variable, *c* is the specific heat capacity, and *Q* represents the power density of the internal heat source per volume. Considering the temperature field as a steady field, we can obtain the following equation:

(2) $$\frac{\partial T}{\partial \tau }=\frac{\partial \theta \left(\tau \right)}{\partial \tau }+\frac{\rho_{w}c_{w}}{\rho_{c}c_{c}}\frac{q_{w}\left[T_{w-in}\left(\tau \right)-T_{w-out}\left(\tau \right)\right]}{V_{c}}$$

where *θ* is the adiabatic temperature rise function of concrete, *ρ_w_*, *c_w_*, and *q_w_* represent the density, specific heat capacity, and water flow of the pipe, respectively, *ρ_c_*, *c_c_*, and *V_c_* represent the density, specific heat capacity, and volume of concrete, respectively, and *T_w-in_* and *T_w-out_* represent the water inflow and discharge of the pipe, respectively. According to the target temperature, we can obtain the theoretical water flow in Equation (3):

(3) $$q_{w}\left(\tau \right)=\frac{\left[T\left(\tau +\Delta \tau \right)-T\left(\tau \right)\right]/\Delta \tau -\theta^{'}\left(\tau \right)}{\rho_{w}\left(\tau \right)c_{w}\left(\tau \right)\left[T_{w-in}\left(\tau \right)-T_{w-out}\left(\tau \right)\right]/\rho_{c}c_{c}V_{C}}$$

**Figure 2:**
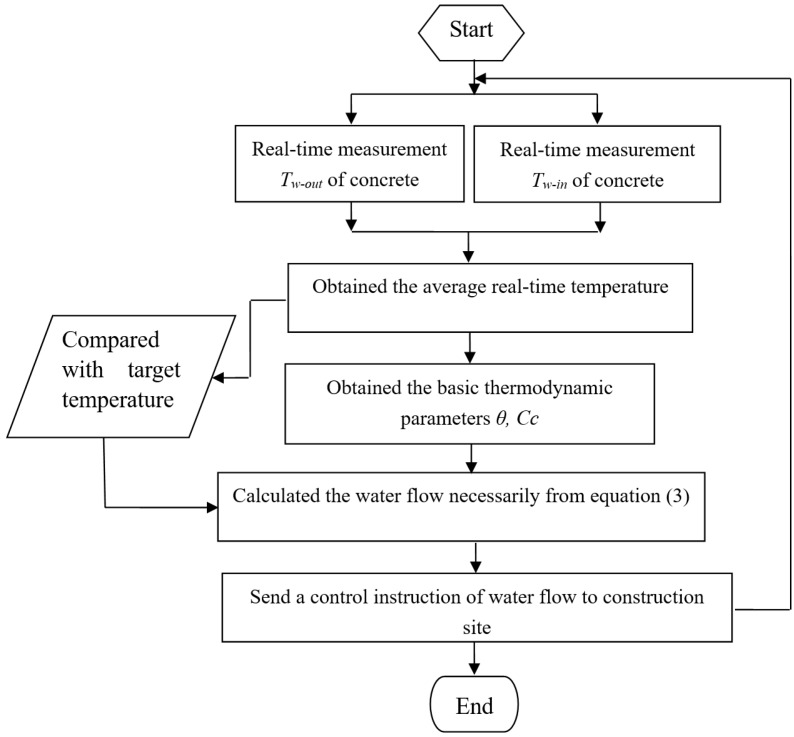
Flow diagram of the concrete temperature control.

Therefore, a basic flow of temperature control, proposed in Figure 2, can be acquired from the above equations.

## 3. Analysis of Electromagnetic Wave Transmission in Concrete

As we all know, concrete is a high-loss material for electromagnetic wave transmitting and, hence, its impact on RFID communication performances should be taken seriously into account. The overall loss of electromagnetic waves penetrating concrete can be divided into two parts: transmission loss and propagation loss [29]. The transmission loss is the power loss resulting from the air-concrete interface and can be defined in dB as:

(4) αt=10×log10(|T|2×Re{η0η1*})

where *η*_0_ is the intrinsic impedance of air and *η*_1_ represents the intrinsic impedance of concrete. Then the propagation loss can be expressed in dB as:

(5) $$\alpha_{p}=10\times \log_{10}(e^{-2ad})$$

where *d* represents the depth of propagation and α is the attenuation coefficient.

**Figure 3:**
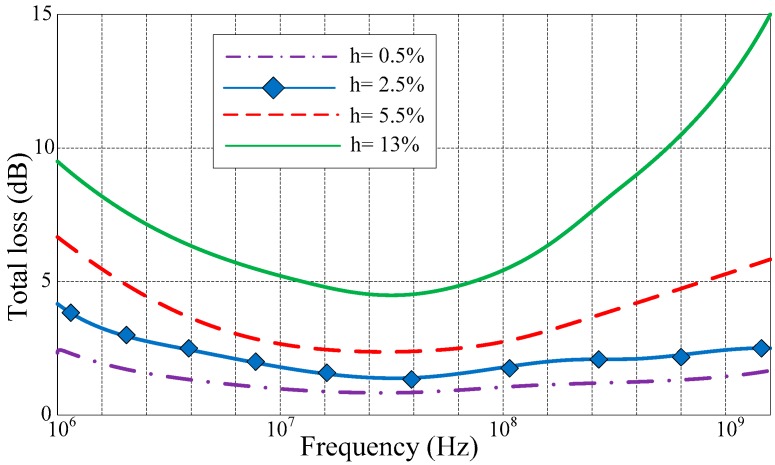
Total loss of electromagnetic waves penetrating concrete.

Since *η*_1_ and α are both highly influenced by humidity conditions of concrete, the transmission loss, propagation loss, as well as overall loss of electromagnetic waves penetrating concrete should be investigated at various concrete humidity conditions. Figure 3 illustrates the MATLAB-simulated results of overall loss at a depth of 0.2 m, from 1 MHz to 1 GHz. The analysis was repeated at typical concrete humidity conditions, i.e., 0.5%, 2.5%, 5.5%, and 13%. The overall loss can be obtained from the sum of the transmission loss and propagation loss. The overall loss of wet concrete (13% humidity) from about 22–82 MHz is about 6 to 11 dB less than the overall loss at either 1 MHz or 1 GHz. Hence, it can be concluded that there should be an optimum frequency with minimum transmission loss from air to concrete. However, the proposed RFID sensor tag works at 915 MHz and, according to Figure 2, the overall loss is about 12 dB.

## 4. Wireless Sensor Tag

### 4.1. Sensor Tag Architecture

**Figure 4:**
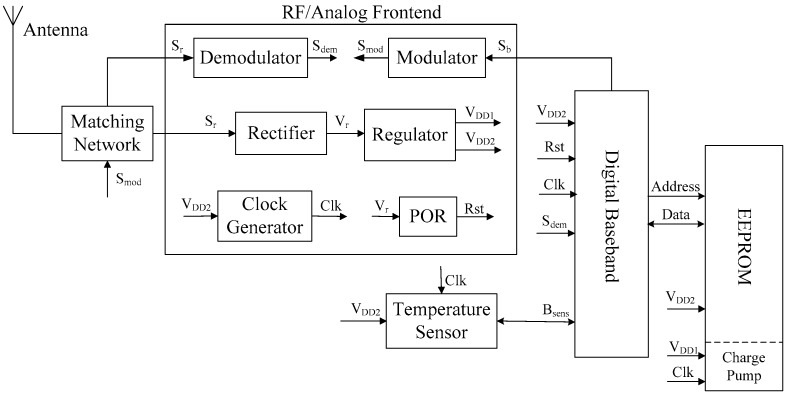
Structure of the proposed RFID temperature sensor tag.

From [23,24,25], the architecture of the proposed RFID temperature sensor tag is shown in Figure 4. The sensor tag is based on the EPC generation-2 UHF communication protocol and operates in passive mode. Except for the antenna and matching network, the other blocks, including the RF/analog frontend, digital baseband, electrically erasable programmable read-only memory (EEPROM), and temperature sensor, are fabricated in a single chip for low-cost application. The sensor tag receives the RF signals from the RFID reader through the antenna and the matching network ensures the optimal point of power matching between the antenna and the tag chip. The RF/analog frontend transfers the received RF signal into stable voltages (through rectifier and regulator blocks) to activate the other internal blocks of the sensor tag. The demodulator (or modulator) block is responsible for demodulating (or modulating) the received (transmitted) signals. The clock generator and Power on Reset (POR) blocks provide the clock and reset signals for digital parts and the sensor interface, respectively. The digital baseband controls the overall operation of the sensor tag according to the EPC generation-2 UHF communication protocol. The EEPROM is responsible for storing the required information, including the EPC code, security data, and sensing data.

The operating power of a RFID tag *P_t_* can be derived from the equation below [30]:

(6) $$P_{t}=P_{r}\times G_{a}\times \eta_{t}\times {\left(\frac{\lambda }{4\pi d}\right)}^{2}$$

where *P_r_* represents the effective radiation power of a RFID reader, *G_a_* represents the gain of tag antenna, *η_t_* is the power conversion efficiency of RF-to-DC of the tag, *λ* is the wavelength of the electromagnetic wave, and *d* is the communication distance between reader and tag. According Equation 6, the communication distance *d* can be described as following:

(7) $$d=\frac{\lambda }{4\pi }\sqrt{\frac{E_{r}G_{a}\eta_{r}}{P_{t}}}$$

Generally, *E_r_* is limited by the RFID communication standards (4 W is the maximum transmitted power) and *G_a_* is approximately decided by the allowable antenna area (1.64 for the *λ*/2 dipole antenna). The communication distance is the most important parameter for designing the RFID tag and guarantees the stability and the reliability of the RFID system. According to Equation (7), in order to achieve higher *d*, lower *P_t_* and higher *η_r_* are critical for the UHF RFID tag design.

### 4.2. Antenna Design

**Figure 5:**
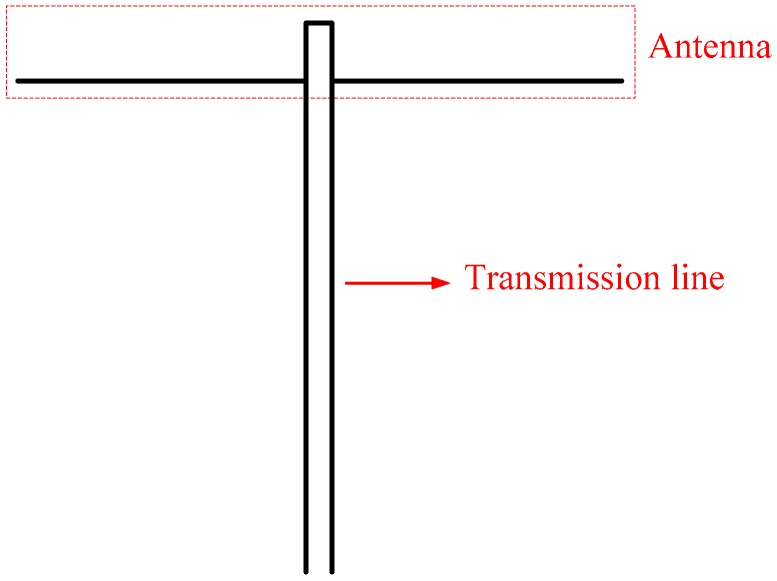
Layout of the proposed T-type antenna.

This work aims to design a RFID sensor tag to perform temperature measurement at the required depth in concrete; therefore, the design of tag antenna should take concrete’s influence on communication into consideration. From the discussion above, the overall loss of 915 MHz at the depth of 0.2 m is about 10 dB, resulting in the UHF RFID tag being hardly read from the external RFID reader. Hence, we proposed a T-type antenna design in Figure 5. Ensuring the low-cost application, the antenna design adopts a simple dipole scheme. In order to enlarge the depth of the tag embedded in concrete, the dipole antenna is connected to an extension part, which consists of two parallel transmission lines made by microstrips [31]. The dipole antenna with a 9.2 cm width is printed on FR4 substrate (dielectric constant *ε_r_* = 4.4, thickness *h* = 2 mm). These transmission lines are designed to transmit the energy collected by the antenna to the inner part of the tag in concrete. The microstrips are insulated from both sides to prevent any degradation of the communication performance. In order to transmit all of the power received by the antenna to the sensor chip, the length of the transmission line must be a multiple of λ/2 in the medium. In this design, the length of the transmission line is 14.2 cm.

### 4.3. Temperature Sensor Design

Generally, the power dissipation of a passive RFID tag is not over 25 µW, the power of the sensor integrated in the RFID tag is always limited to under 5 µW. Hence, the power of the sensor is the key for the integration between the RFID tag and the sensor [15]. Furthermore, as for the RFID tag, the internal clock generator always has low accuracy for low-power and low-cost applications, resulting in the low-accuracy of the integrated temperature sensor using this clock. The traditional CMOS temperature sensors [32,33] always use two steps: temperature-voltage conversion and voltage-digital conversion. However, these sensors adopt complicated architectures and have to work under high supply voltages, resulting in high power consumption. Recently, various low-power design schemes, such as digital conversion in the frequency domain [34] or time domain [35,36], have been introduced to achieve ultra-low power dissipation for wireless applications. However, these designs still have to employ external reference clocks.

**Figure 6:**
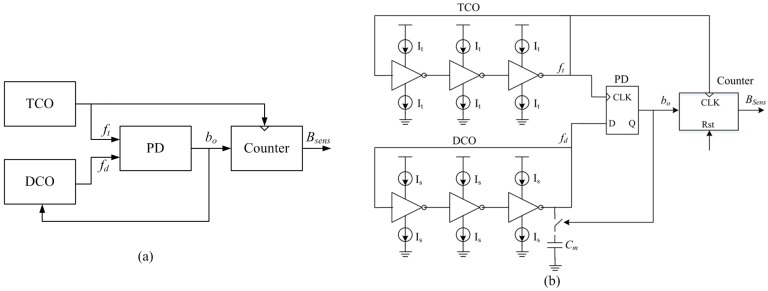
Proposed temperature sensor: (**a**) architecture; and (**b**) schematic.

The architecture of the proposed temperature sensor is shown in Figure 6a. It developed from the phase-locked loop (PLL)-based interface theory [37] and can be divided into four blocks: a temperature-controlled oscillator (TCO), a digitally-controlled oscillator (DCO), a phase detector (PD), and a counter. The TCO is responsible for converting the temperature information into a temperature-controlled frequency *f_t_*. The DCO is used to generates a digital-controlled frequency *f_d_*, which is controlled by the PD’s output *b_o_*. PD detects the phase difference between the TCO and DCO and then generates a corresponding digital signal *b_o_*. PD combines with TCO and DCO to form a PLL loop. The counter, utilizing *f_t_* as the reference clock, is responsible for converting *b_o_* into a corresponding digital code *B_sens_*. When the entire loop is stabilized, *B_sens_* represents the digital value of the temperature information.

The schematic of the proposed temperature sensor is shown in Figure 6b. Both the TCO and DCO employ an inverter-based ring-oscillator structure. The frequency of invert-based oscillator *f_osc_* can be expressed as:

(8) $$f_{osc}=\frac{1}{t_{d}}=\frac{I_{l}}{V_{m}C_{l}}$$

where *t_d_* is the delay time of the loop, *I_l_* is the current flowing through the inverter, *C_l_* is the equivalent load capacitor of the loop, and *V_m_* is the swing range of the output voltage which mostly equals the supply voltage *V_DD_*. According to Equation (8), each inverter of the TCO adopts a proportional to absolute temperature (PTAT) current source *I_t_*, resulting in the frequency of TCO *f_t_* being a PTAT frequency. Different from the TCO, the current source of DCO *I_t_* is set equal to the quiescent part of *I_t_*, the resulting *f_d_* equals the quiescent part of *f_t_* when the capacitor *C_m_* is connected to the DCO loop. The *C_m_* connects or not to the DCO loop depending on the output *b_o_* from the PD. The value of *C_m_* is designed to make *f_d_* slightly larger than *f_t_* when *C_m_* connects to the DCO loop.

### 4.4. Rectifier Design

**Figure 7:**
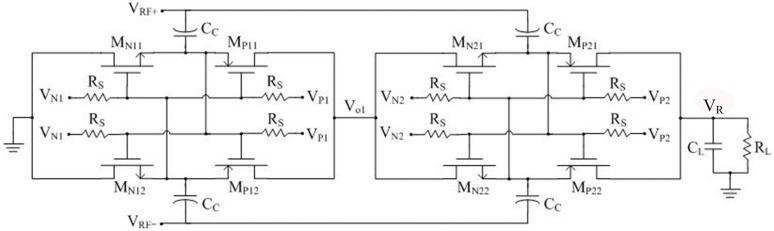
Schematic of the proposed rectifier.

Inspired by Kamalinejad [38], a gate-boosting scheme is adopted to improve the power conversion efficiency *η_r_* of the rectifier. When the input power is smaller (or larger) than the optimal point of the *η_r_* curve, an extra bias-voltage can be added positively (or negatively) to promote the effective gate-source voltage of the switches. In addition, the extra bias-voltage equals zero when the input power comes at the optimal point, which means the bias-voltage is dynamic. From the above rules, Figure 7 shows the proposed rectifier is made up of two identical stages. The differential-drive switch is constituted by the NMOS transistors M_N11-22_ and PMOS transistors M_P11-22_, the gate-source voltage of which are boosted by the bias-voltage *V_N_*_1*,*2_ and *V_P_*_1*,*2_, respectively. The large resistor *R_S_* is adopted to block the AC component of *V_N_*_1*,*2_ and *V_P_*_1*,*2_. In order to avoid the large silicon area in the CMOS process, a PMOS transistor which operates in the cut-off region could replace *R_S_*.

## 5. Experimental Characterization

**Figure 8:**
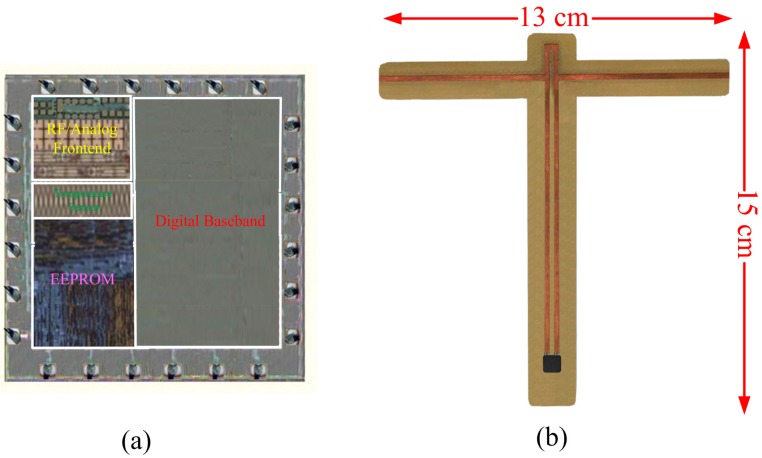
Photo of the proposed RFID sensor tag: (**a**) tag chip; (**b**) RFID sensor tag.

Figure 8a shows the microphoto of our RFID sensor tag chip. The RFID tag chip was designed and fabricated using the TSMC 0.18 μm 1P6M mixed-signal CMOS process. As shown in Figure 8b, this tag chip was matched with the proposed T-type antenna on FR4 substrate for the temperature measurement in the laboratory. The overall tag covers an area of approximately 13 × 15 cm^2^.

**Figure 9:**
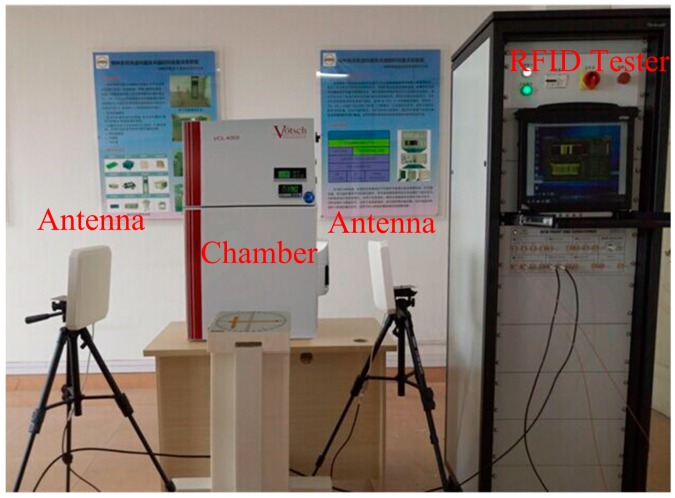
Wireless measurement environment.

The sensor tag was firstly tested and calibrated in the laboratory, as shown in Figure 9. In order to improve the measuring accuracy, two antennas were adopted in our measurement, one was the transmitting antenna and another was the receiving antenna. The temperature calibration of the tag was performed inside a VCL4003 temperature and humidity chamber from Votsch Industry Electronics (Taichang, China). The VISN-R1200 from VI Service Network (Shanghai, China) is an RFID comprehensive test instrument from VI Service Network (Shanghai, China), which can assist in research, development, and measurements based on various RFID standards.

**Figure 10:**
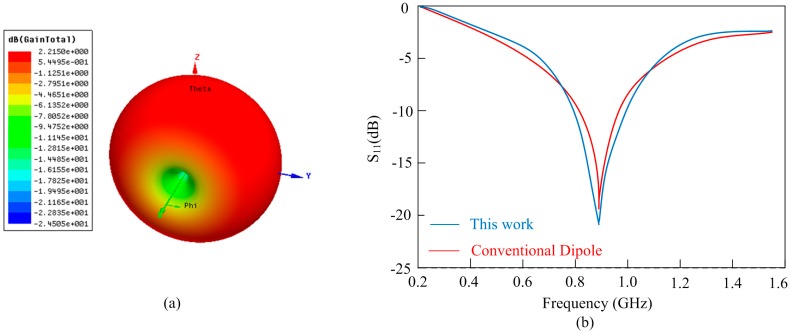
Antenna performances: (**a**) radiation pattern; (**b**) S_11_ performance.

The performances of the proposed antenna is shown in Figure 10. The E-plane polar diagram in Figure 10a illustrates the simulated radiation pattern performances and the maximum realized gain is 2.21 dB. In order to compare with the conventional dipole, a similar antenna, only without transmission lines, is connected the proposed sensor chip for the measurements. From Figure 10b, these two measurements show a good coincidence. Therefore, due to the transmission line, the proposed T-type antenna can efficiently extend the embedded measuring depth in concrete.

**Figure 11:**
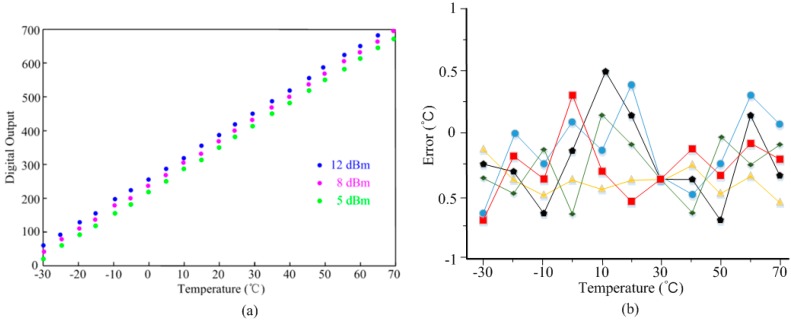
Tested performances of the temperature sensor: (**a**) linearity; and (**b**) error.

In order to test the temperature performances, the proposed sensor tag is put into the climate chamber together with an antenna of the RFID tester. The measured digital outputs of the tag at different input powers (12, 8, and 5 dBm) are shown in Figure 11a. The digital output of the tag can read from the reader. The three tests show high consistency and achieve a linearity error of less than 6%. The measured resolution is around 0.15 °C/LSB within the temperature range of −30–70 °C. Figure 11b illustrates the temperature error performances of five different test sensor tag chips. All five of the test chips are calibrated at 30 °C by adjusting the capacitance of *C_m_*. Compared with [23,24,25], the measured temperature sensor achieves a higher temperature error of −0.7/0.5 °C within the range of −30–70 °C due to the temperature sensor design lacking a reference clock signal.

**Figure 12:**
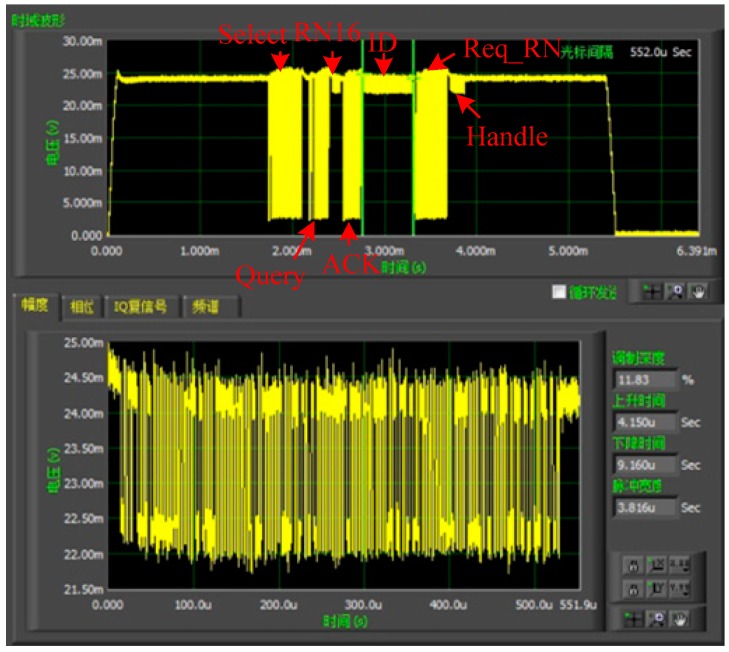
The result of communication test in the laboratory.

To verify the RFID communication of the proposed sensor tag, the testing parameters are chosen as follows: the operating frequency is 915 MHz, the distances between the two antennas and the sensor tag are both 0.5 m, the transmitting power is 1 W. The overall communication flow is shown in Figure 12. The RFID tester firstly sends a Select Instruction of Inventory Sequence order. After waiting for 5~6 Tari, the RFID tester then sends a Query instruction and the tag responses back with RN16. The tester then sends an ACK instruction to acquire the ID information of the tag, including the sensor data. After that, the tester sends Req_RN instruction to obtain the Handle response.

**Figure 13:**
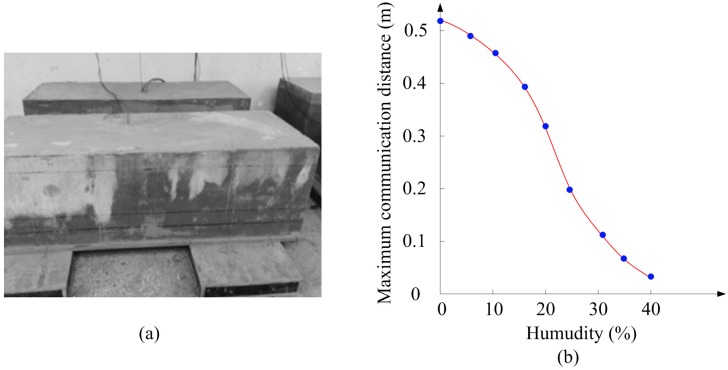
Maximum communication distance at 915 MHz: (**a**) test environment; (**b**) test results.

The exploited RFID temperature sensor tag is buried in concrete for temperature monitoring, so the transmission loss of the sensor tag under different humidity conditions significantly influences its performance. Thus, the transmission loss under different humidity conditions is experimentally measured. A Sensirion SHT75 humidity sensor is employed to measure the concrete humidity. As is shown in Figure 13a, the experimental concrete block is 120 × 30 × 30 cm^3^ and this block is composed of 50% ordinary Portland concrete and 50% sand. The SHT75 humidity sensor and the proposed RFID temperature sensor tag are all buried in the concrete, then a handheld RFID reader is placed on the surface of concrete to measure the maximum communication distance between the reader and the antenna of the sensor tag under different humidity conditions. If the successful reading ratio is more than 80%, the communication distance can be considered an effective communication distance. The measurement results are shown in Figure 13b. It can be seen that a negative correlation between humidity and maximum communication distance appears.

**Figure 14:**
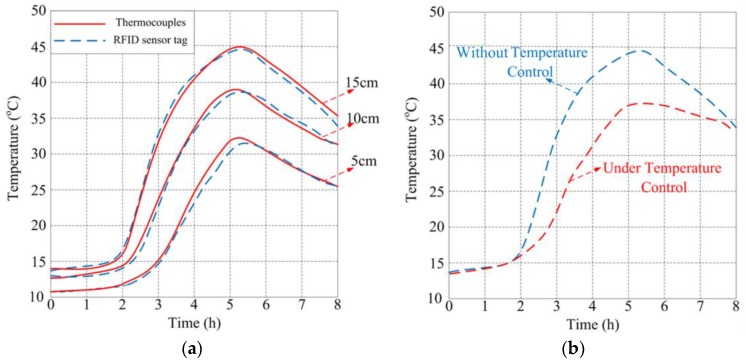
Temperature performance measurement of concrete: (**a**) measured internal temperature of concrete versus time in two different ways; and (**b**) comparison between testing under temperature control and without temperature control.

The temperature performances of the concrete during the maturation process were tested by the proposed RFID sensor. The tests were repeated when the RFID sensor was immersed at depths of 15, 10, and 5 cm, respectively. Three additional thermocouples were, respectively, placed in the same location as the references. Two hours after casting, the specimen used for testing was heated by an oven to the temperature of 50 °C for three hours, then the oven was turned off for the analysis of the cooling process of the concrete. During the heating and cooling process, the temperature was measured by both the thermocouples and the proposed RFID temperature sensor tag. The temperature measured results without intelligent control are shown in Figure 14a; it is clear that the two curves follow nearly the same profile and the maximum difference is less than 0.5 °C. Due to the internal curing process, the shallower site is easier to radiate heat, resulting in the temperature in the deeper site being higher than the shallower site. The comparison between testing under temperature control and without temperature control on the site at a 15 cm depth is shown in Figure 14b. It is obvious that the temperature under intelligent control rises and declines more gently than the temperature without control.

**Figure 15:**
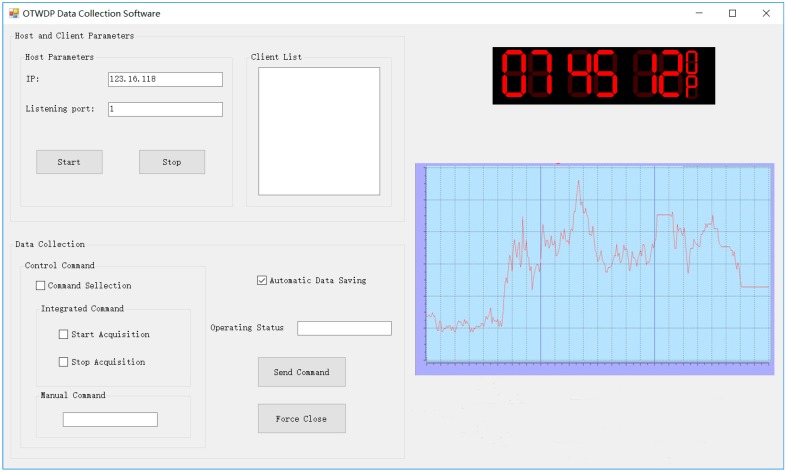
Software interface of the concrete temperature monitoring system.

The monitoring software interface for concrete temperature is shown in Figure 15. This software interface mainly consists of four parts: a host and client parameters module, a data collection module, residual error display module, and an alarm module. The host and client parameters module is used to configure and display local and remote parameters, such as IP, listening port, and client list. The data collection module is used to select the command type or enter manual commands. The temperature display module is used to display the residual error value and curve. The alarm module is green when the temperature is within the normal range, and changes to red when the temperature is abnormal, making it easier to be identified.

The proposed non-destructive concrete temperature monitoring system can track the temperature inside the large volume concrete in real-time, compared with traditional methods, it reduces the cost and avoids a complex layout. Table 1 compares the measured performances of the proposed method with other concrete temperature monitoring methods. TTM and FTD have complex layouts, low flexibility, and high maintenance costs, so they are not suitable for large-scale commercial applications. The temperature measurement based on WSN technology decrease the cost and has a simple layout. Additionally, the maintenance cost is also decreased, but it requires a battery as a power supply. The proposed RFID-based technique takes advantages of the WSN-based method and it does not need an additional battery for power.

## 6. Conclusions

Temperature monitoring plays a dominant role in concrete measurement. In this work a wireless temperature sensor tag based on the EPC generation-2 UHF communication protocol is proposed to monitor the temperature inside concrete online. Considering the high losses of electromagnetic waves in concrete, a T-type antenna is proposed to ensure the sensor tag can work inside the concrete. The temperature sensor employs a PLL-based architecture and can transfer the sensor values to a digital output in the frequency domain without a reference clock. The measured results show the proposed sensor tag achieves high temperature linearity. The communication flow of the proposed sensor tag was tested in the laboratory, the maximum operating distance and the temperature measurement were tested on a construction site. The measured temperature results by the RFID sensor and thermocouple are almost consistent. The whole experiment demonstrates that the proposed sensor tag can provide reliable performance in wireless concrete temperature measurement.

## Figures and Tables

**Table 1:** Performance comparison of different concrete temperature monitoring methods.

Methods	Convenience	Real-Time	Cost	Life Time	Accuracy
TTM	moderate	good	moderate	moderate	good
FTD	disappointing	good	high	moderate	outstanding
WSN	outstanding	outstanding	low	moderate	moderate
This work	outstanding	outstanding	ultra-low	long	moderate
